# Perceptions of Dementia among Nursing Students in South Korea: A Phenomenological Study

**DOI:** 10.3390/healthcare12161565

**Published:** 2024-08-07

**Authors:** So-Hee Lim

**Affiliations:** Department of Nursing, Kyungmin University, Uijeongbu-si 11618, Republic of Korea; sweetnurseme@naver.com; Tel.: +82-31-828-7472

**Keywords:** dementia, perception, nursing student, empathy

## Abstract

This study aimed to determine nursing students’ perceptions of dementia and patients with dementia. Specifically, this study sought to illuminate the essential meaning of dementia among nursing students to uncover insights useful for developing patient-centered care rooted in empathy for patients with dementia. This study used a descriptive phenomenological research method. Between August and September 2023, semi-structured interviews were conducted with 15 nursing students. Colaizzi’s method was used for the manual analysis. After analyzing the interview data on nursing students’ awareness of dementia, four categories, nine theme clusters, and 28 formulated meanings were derived. The four categories were “View of dementia”, “Feelings about dementia”, “Efforts made regarding dementia”, and “Dementia as a nursing student”. Although nursing students’ perceptions of dementia resulted in negative perspectives and emotions, by looking into coping methods and wishes for dementia, this study also confirmed that nursing students had an awareness of caring for patients with dementia.

## 1. Introduction

Amid recent advancements in medical technology and increased life expectancy, South Korea is now considered an “aging society”, whereby the 65+ years old population accounts for a large percentage of the total population. Although these older adults accounted for only 7.2% of the population in 2000, that percentage has now risen to 14.3% as of 2018. Furthermore, it rose to 15.7% in 2020 and is projected to rise to 20.3% in 2025 [1,2]. In addition to the increasing older adult population in South Korea, the prevalence of various age-related diseases has also risen. Specifically, the number of patients with dementia in 2020 was estimated at approximately 860,000; this figure is expected to increase to 1.36 million by 2030, 2.2 million by 2040, and over 3 million by 2050 [1].

Dementia is a syndrome caused by chronic or progressive brain diseases and involves multiple impairments in brain cortical functions, including memory, thinking, comprehension, calculation, learning capacity, language, and judgment. Generally, the prevalence of dementia increases with age [2,3]. In other words, dementia, a degenerative neurological condition that progresses over time, leads to cognitive impairment and behavioral disorders, hinders independence and fulfillment of social roles, and thus requires long-term care and support from family or society [1,2,4].

In response to these issues, the South Korean government enacted the Dementia Management Act in August 2011 and has focused on expanding dementia-related infrastructure, including the CDC, regional dementia centers, dementia support centers, and dementia support hospitals. The South Korean government has strived to strengthen age-specific dementia management from the perspective of those affected and to realize a dementia-friendly society involving patients, families, and communities. Subsequently, a national responsibility system for dementia and a linked system for dementia-related infrastructure and foundational policies through systemic improvements were established in September 2020 [1]. Despite these governmental efforts, negative perceptions of dementia, patients with dementia, and dementia care remain prevalent.

In Chinese characters, the word “dementia” is a combination of the words “foolishness” (癡) and “stupidity” (呆), revealing the inherently negative connotations of the word in East Asian cultures. In mass media, including broadcasts and news, patients with dementia are often depicted negatively, which has contributed to the formation of prejudiced and stereotypical views [5,6]. Surveys in South Korea have demonstrated an increase in negative perceptions toward dementia, rising by 4.2 percentage points from 39.6% in 2014 to 43.8% in 2021, indicating an urgent need to address these perceptions. One of the reasons for these negative views was the fear of dementia (60.2%) [1], highlighting the need for awareness-raising and educational activities about dementia. Additionally, demographic changes owing to rapid aging and a shrinking workforce are causing intergenerational issues, with a clash of economic interests between generations leading to intergenerational conflicts. Therefore, negative perceptions of dementia among younger generations must be improved.

College students—a prime example of the likely younger generation—will not only be the primary caregivers for older adults in the future but also the most directly affected by the societal issues related to aging and dementia. The values and attitudes cultivated during college lay the foundation for their social life and future, and their perceptions of dementia are crucial. This is particularly true for nursing students, as these prospective nursing professionals will directly interact with patients with dementia and their caregivers in healthcare settings in the future. Hence, nursing students must be equipped with well-informed perceptions, positive attitudes, and professional knowledge about dementia [7,8]. Nursing students must be prepared to provide care based not only on pathological knowledge of the disease and its treatment but also on a psychological and emotional understanding of those affected by dementia. Many nursing students in South Korea are experiencing reduced empathy and understanding toward others due to stress from clinical rotations and academic workload, competitive environments, and self-centered thinking [9]. Moreover, nursing education on dementia in South Korea is heavily theory-oriented, limiting the fostering of understanding and empathy toward patients with dementia [10]. Therefore, to enhance positive perceptions and attitudes toward dementia among nursing students, this study analyzes their understanding and perceived meaning of dementia.

Qualitative research emphasizes the meaning of a phenomenon and the experiences or views of the subjects, deriving concepts that help understand the phenomenon in its natural context. Phenomenology is one of the representative qualitative research methods. Phenomenology promotes understanding of humans and explains the nature of behavior based on meditative thinking [11]. In other words, phenomenology uncovers the meaning of phenomena experienced by humans through an analysis of the subject’s description. In the nursing context, phenomenological work can enable nurses to increase their impact by strengthening their awareness of others and exploring diverse perspectives through engaging in careful descriptions and dialogues [12]. The phenomenological research process involves closely reading and re-reading descriptions to uncover the lived meanings of research participants, which are derived from both implicit and explicit messages in the data. This research focused on uncovering nursing students’ perceptions of the meaning of dementia. Given this aim, this study selected the phenomenological research approach, which enables exploring and reflecting on the inner worlds of participants and describing the meaning of their experiences and perceptions by understanding the essence of phenomena. Thus, this study provides an in-depth understanding of nursing students’ interpretations of dementia. The present results may help lay the groundwork for developing tools to measure nursing students’ perceptions of dementia and create appropriate educational programs. Ultimately, this study seeks to enhance the quality of care for individuals with dementia.

## 2. Materials and Methods

### 2.1. Design

This study used a descriptive phenomenological approach informed by the philosophy of Colaizzi [11]. We chose this approach because Colaizzi’s philosophy recommends bracketing to exclude personal preconceptions and assumptions. Furthermore, it advocates intuiting, encouraging individuals to focus on the pure existence of phenomena to establish the fundamental intentionality of consciousness [11]. This could help provide a deep and essential perception among nursing students.

### 2.2. Preparation of the Researcher

The researcher of this study had completed courses related to qualitative research during their doctoral program and has been attending related conferences and seminars at least once a year. They have consistently engaged in exploratory activities in the field and have recently conducted and published a qualitative study. Additionally, the researcher has thoroughly read books on phenomenology and published qualitative studies to deepen their understanding of the research methods. Furthermore, they are committed to enhancing their qualitative research skills and strive to expand their perspective for grasping the essence of phenomena.

### 2.3. Participants

This study recruited college students currently enrolled in nursing programs within the Seoul metropolitan area. This study selected nursing students based on the assumption that they would be most capable of effectively expressing insights on dementia, the focus of this phenomenological research. Recruitment announcements were posted on websites and bulletin boards. Additionally, individuals who provided informed consent to participate were recommended and conveniently sampled as needed. This study utilized purposive sampling to select participants who could provide in-depth and relevant information appropriate to this study’s theme and objectives. This study enrolled 15 nursing students from four nursing schools who had experience meeting patients with dementia, including 4 men and 11 women. They comprised three first-year students, three second-year students, five third-year students, and four fourth-year students. Data were collected until saturation.

### 2.4. Data Collection

The data were collected via in-depth interviews from August to September 2023. Before the interviews, the researcher explained the purpose of this study, data collection method, and audio-recording protocol to the participants. After the participants provided informed consent, they were asked to sign a written consent form. The interviews were held in a private, quiet place where the participants could feel at ease. The key questions of this study were “What do you think about dementia?”, “What do you think are the implications of dementia?”, and “What are important aspects of providing care for patients with dementia?”. Follow-up questions were asked based on the participants’ responses for an in-depth exploration. During the interviews, non-verbal cues, such as gestures, facial expressions, and tones, were also observed and recorded. Each interview lasted approximately an hour. After the interviews, any unclear parts in the interview transcripts were clarified through phone calls or social media messaging. The interviews continued until data saturation, when their responses began to repeat, and no new information emerged. Data collection in qualitative research prioritizes not the sample size but rather the usefulness of the resource [13]. Data collection is complete when a state of theoretical saturation is reached, in which no new concepts or categories can be derived.

### 2.5. Ethical Considerations

This study was reviewed and approved by a joint institutional review board (no. P01-202308-01-028). The participants were provided an explanation about the study purpose and procedure and audio-recording of the interviews. A written informed consent form was obtained from those who voluntarily consented to participate in this study. The participants were informed that the interview data would only be used for research purposes and that personal information would be removed to guarantee anonymity. Those who consented were enrolled in this study. The participants were also informed that they had the freedom to withdraw from this study at any time and were provided a detailed explanation of the study process to protect their rights. Moreover, the researcher excluded certain statements that the participants requested not to be published in the article to respect their wishes.

### 2.6. Rigor

The researcher ensured the rigor of this study using the criteria of credibility, transferability, dependability, and confirmability [11,12]. For credibility, the researcher validated the transcribed interview data by having participants review them. Furthermore, the analysis results were presented to them to confirm their accuracy. To achieve transferability, the researcher collected data through in-depth interviews until no new information emerged. Additionally, the researcher received feedback on the study findings from three college students not involved in this study. To ensure dependability, the researcher strictly adhered to Colaizzi’s [11] study method and analysis procedures. During the data analysis, the researcher consulted with two qualitative researchers to verify the accuracy of the conceptual statements. The entire study process was reviewed to reach a consensus on the results. To maintain confirmability, the researcher used structured questions during the in-depth interviews, endeavored to suspend personal judgments, and refrained from critiquing the content during the analysis process. Furthermore, the researcher received feedback from two qualitative researchers to check whether the researcher’s subjective thoughts influenced the interpretation process. Credibility was maintained in a state of “suspension of judgment” to exclude the researcher’s preconceptions and prejudices. Additionally, continuous reflection was conducted while analyzing the research results to understand the participants’ perspectives.

### 2.7. Data Analysis

**Figure 1:**
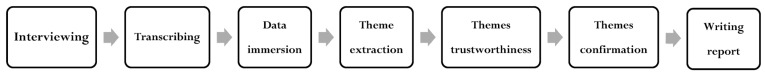
Phases of the method.

In this study, the data collection and analysis were conducted simultaneously and cyclically, following the phenomenological research method outlined by Colaizzi [11,12,13]. In the first stage, the researcher transcribed the interview data from the recorded sessions, referring to notes taken during the interviews. This process helped the researcher gain an overall impression and understanding of the general meaning of the participants’ perceptions of dementia. By repeatedly reading the original data, the researcher grasped a comprehensive feeling of the participants’ perceptions of dementia. Next, the researcher carefully read the transcribed content to identify and extract meaningful statements from recurring sentences or expressions. These meaningful statements were then articulated in a more general form in the third stage. Following this, meanings were derived from these statements and rephrased in the researcher’s language while ensuring their context matched the original data. In the fifth stage, the constructed meanings were analyzed and categorized into categories, theme clusters, and formulated meanings. Finally, the researcher connected the themes with the phenomenon to produce a comprehensive and final description (Figure 1).

## 3. Results

Regarding nursing students’ perceptions of dementia, this study identified four categories, nine theme clusters, and 28 formulated meanings. The four categories were “View of dementia”, “Feelings about dementia”, “Efforts made regarding dementia”, and “Dementia as a nursing student” (Table 1).

### 3.1. View of Dementia

In the first category, nursing students shared their perceptions of dementia and patients with dementia based on their thoughts and experiences. This category revealed a negative perception of dementia, with “Illness” and “Suffering” emerging as theme clusters.

#### 3.1.1. Illness

The participants viewed dementia as an incurable disease and an illness that must be avoided. Furthermore, they perceived dementia as a type of disease.

*It’s a scary, sad, and lonely illness. It’s hard to even imagine. *

*(Participant 2)*

*You never know when you’ll get dementia, and the thought of facing this worst possible scenario is despairing. There’s no cure, and I think I’d be having a hard time every moment. *

*(Participant 3)*

*Dementia isn’t a disease to be hidden; it’s a brain disorder. It requires treatment and ongoing management. *

*(Participant 5)*

#### 3.1.2. Suffering

The participants perceived dementia as an affliction that erodes human dignity, causing suffering not only for the individual but also for their families by destroying families and causing economic problems.

*Living with dementia means losing the ability to understand the world and express oneself. It seems incredibly hard not to be able to do anything by yourself and to lose the ability to perform daily life functions. *

*(Participant 1)*

*My late grandfather had dementia. He often got lost and was picked up by the police, and it caused my parents a lot of stress. His emotional fluctuations became extreme, and it made it difficult for my parents to work, eventually leading our family to a tough situation and placing him in a nursing home. *

*(Participant 8)*

*I want to care for my parents myself. But I wouldn’t know what to do if I’m not financially stable or can’t quit my job. *

*(Participant 14)*

### 3.2. Feelings about Dementia

The participants expressed their negative emotions about dementia. They described their feelings toward dementia based on direct care experiences, indirect experiences through family or close relatives caring for a person with dementia, and contemplations about themselves or family members potentially being diagnosed with dementia in the future. This category included the theme clusters of “Distant feeling”, “Fear”, and “Feeling sorry”.

#### 3.2.1. Distant Feeling

The participants described dementia as unrelated to them and something that could never happen to them. They expressed vague emotions toward people with dementia.

*I don’t have any thoughts about dementia. It’s a disease unrelated to me, and no one in my family has it. My parents and grandparents are all healthy. I’ve heard stories from others, but it seems like it’s not my issue. *

*(Participant 5)*

*If I were to get dementia, it feels like something that would never happen to me. *

*(Participant 8)*

*I think we need to think that, “I could also get dementia someday”. I keep thinking, “Would I be okay with someone treating me the way I treat patients with dementia?” *

*(Participant 12)*

#### 3.2.2. Fear

The participants expressed fear about memory loss and abnormal behaviors associated with dementia. They stated that dementia is an illness that occurs due to aging. Moreover, they expressed negative feelings regarding being diagnosed with dementia themselves or having a family member diagnosed and becoming a caregiver.

*Memory loss is truly frightening. It would feel like being left alone in the world as you can’t recognize anyone, with your memory being lost gradually. *

*(Participant 4)*

*Dementia is related to memory loss, leading to delusions, increased aggression, sleep disorders, incontinence, and inability to carry out daily activities. If I were to develop dementia, I wouldn’t be able to carry on with my social life anymore. *

*(Participant 6)*

*I think people become more likely to develop dementia as they age. People are afraid of and worried about dementia. It’s not just hard for the person with dementia but also for those around them, so I have a vague fear of dementia. *

*(Participant 9)*

*I think people who care for a patient with dementia might be more mentally than physically exhausted. While physical fatigue can be relieved through hobbies or meeting friends, mental stress doesn’t easily go away. But more than anything, caring for a loved one with dementia might be not too hard at first but would become increasingly difficult as they lose their bodily functions and you have to provide total care. It would be mentally and physically challenging. The pressure of needing to care for them can also be overwhelming. *

*(Participant 11)*

#### 3.2.3. Feeling Sorry

Owing to a lack of direct nursing experiences with dementia, the participants shared their third-person experiences regarding patients with dementia and caregivers and reflected on how their attitudes toward dementia were becoming more negative.

*I volunteered to take care of patients with dementia. One older man ate soil in the garden, and another repeated the same behaviors. They had no visitors and just stayed in the hospital all day. Dementia seems like a very lonely disease. *

*(Participant 3)*

*I saw an older man taking care of his wife with dementia. He did everything by himself because he didn’t want to pass on the burden to their adult children. The older man also had aches everywhere. *

*(Participant 9)*

*I met an old lady with memory issues. She kept asking the same question every five seconds, and it was awkward for me to keep answering the same thing. Even after having lunch, she forgot about it and threw tantrums every 30 min, asking for food. Every moment of taking care of her was extremely challenging for me. *

*(Participant 13)*

### 3.3. Efforts Made Regarding Dementia

The participants proposed strategies to address dementia for individuals with dementia, caregivers, and society. They discussed the care provided by families, social care, and the potential for future treatments. The theme clusters in this category were “Strategies to address dementia” and “Hopes”.

#### 3.3.1. Strategies to Address Dementia

The participants’ perceptions and feelings about dementia were not purely negative; they also spoke about their roles as nursing students in preventing dementia and dealing with people with dementia, as well as the role of society.

*To prevent dementia, regular exercise, a healthy and balanced diet, daily cognitive stimulation through activities like diary writing, puzzles, and maintaining hobbies are essential. *

*(Participant 6)*

*Even if I don’t have dementia and it’s not something I see around me, it could happen someday, so it’s important to learn about dementia. We should try to understand them, accept them, and treat them as equals.*

*(Participant 12)*

*As society ages, the prevalence of dementia is increasing and will continue to do so. In an aging society, it’s crucial for the community and society as a whole to collaboratively provide the care and support needed for patients with dementia to maintain their independence and quality of life. *

*(Participant 15)*

#### 3.3.2. Hopes

The participants expressed hopes for changes and improvements in family care and various social care services for people with dementia, as well as possibilities for future treatments.

*If my parents develop dementia, it’s important to support them in everything they can do. Since dementia cannot be overcome by patients alone, I think all members of the family need to collaboratively help and support them. You should babyproof all furniture and corners and will need to engage in activities like dementia prevention quizzes or puzzles together. *

*(Participant 4)*

*I can’t do this alone, so I will seek support from the extensive supportive network, such as healthcare services, support groups, and community organizations, to adapt to dementia. *

*(Participant 10)*

*I hope that caregiving robots or cures for dementia are developed before I develop dementia. *

*(Participant 12)*

### 3.4. Dementia as a Nursing Student

The participants expressed their perceptions of dementia from the perspective of nursing students. As future nurses, they emphasized the importance of providing the best physical and emotional care to individuals with dementia and the need for specialized education in dementia care. The theme clusters in this category were “Sense of duty” and “Need for education”.

#### 3.4.1. Sense of Duty

The participants felt a strong sense of duty to provide the best care to individuals with dementia, treating them with the same respect and attention they would provide their grandparents.

*Like my parents took care of my grandmother, I believe we need to care for individuals with dementia just as if we are caring for family. *

*(Participant 1)*

*Even though dementia may not be curable, I feel I should provide nursing care that helps maintain their current state. It’s not just about performing nursing care but also about providing mental and spiritual care. *

*(Participant 5)*

*Dementia is a geriatric disease, so there’s no end in sight, and tasks are repeated every day. But with continuous advancements in medical technology, we would be able to provide nursing care to maintain their current state. We must take care of them tirelessly and to the best of our ability.*

*(Participant 10)*

#### 3.4.2. Need for Education

As nursing students, the participants expressed the need for specific and professional education about effective nursing care in terms of both skills and understanding the psychology of patients with dementia.

*Dementia is a geriatric disease, and rather than considering it a brain issue, we need actual, professional nursing education about the pathophysiology of dementia, assessment methods, diagnosis, and effective nursing techniques. *

*(Participant 4)*

*We need patient- and field-centered education that can be applied in practice and meet patient needs instead of only learning about theory. *

*(Participant 8)*

*Dementia involves not only physical but also psychological issues, so education in these aspects is necessary. It would be beneficial to understand the psychology of older adults or patients with dementia. *

*(Participant 13)*

## 4. Discussion

This study attempted to shed light on nursing students’ perceptions of dementia through a phenomenological approach. Through analysis, this study derived four categories, nine theme clusters, and 28 formulated meanings. The “View of dementia” category included “Illness” and “Suffering” as theme clusters. The participants perceived dementia as an incurable illness that should be prevented. They noted that individuals with dementia gradually lose their dignity, and their families face economic problems arising from continuous care and treatment costs, potentially leading to family breakdown, which indicated a negative perception of dementia among the participants. According to a 2021 study by the CDC, negative perceptions of dementia are on the rise.

Moreover, nursing students have been reported to have more negative perceptions and attitudes toward older adults with dementia than the general older adult population [14,15], suggesting a widespread negative perception of dementia among nursing students as well. This may be attributable to the fact that dementia is an irreversible disease that makes recovery difficult [15]. Moreover, having vague information about dementia seems to exacerbate fear. A common misconception is that dementia is a normal part of aging, and many people fail to distinguish between early dementia and aging-related cognitive decline [16]. In other words, while the participants perceive dementia as an abnormal state and illness caused by a decline in brain functions, they believe that the illness itself is a natural part of aging. Considering the finding that inaccurate perceptions and knowledge of dementia can lead to a negative outlook on the condition, Kim and Chang [2] highlighted the need to enhance the knowledge and understanding of dementia through specialized education tailored for nursing students to foster a positive perception.

Furthermore, accurate and comprehensive information about dementia should be provided to positively transform the perceptions of dementia and improve the social atmosphere, which would lead to better dementia prevention, early detection, and proactive social support systems. In addition to the negative perceptions, previous studies have also reported positive aspects, such as enhanced self-efficacy, personal growth, pride, maintaining intimate relationships, and job satisfaction from caring for individuals with dementia [17,18,19]. Therefore, future studies should perform a detailed analysis of factors that could improve positive perceptions of dementia.

The second category, “Feelings about dementia”, included the theme clusters of “Distant feeling”, “Fear”, and “Feeling sorry”. Our participants perceived dementia as irrelevant and unlikely to happen to them; the predominant feeling when considering the prospect of either developing dementia themselves or providing care to individuals with dementia was fear, similar to previously reported findings [7,8,19]. Additionally, our participants’ emotions toward dementia tended to become more negative through direct or indirect caregiving experiences. Previous studies [7,8] observed that nursing students tend to express aversion and a sense of burden toward caring for individuals with dementia, and Yeom [19] reported that nurses in long-term care facilities feel a sense of distance, fear, and barriers when dealing with patients with dementia.

Furthermore, family caregivers of patients with dementia face issues such as stress, depression, and a sense of burden, and as caregiving is prolonged, conflicts arise among family members as well as financial burdens [18,20]. Scholars have reported that caregiving may even lead to suicidal ideations and suicide attempts [21]. Negative emotions, such as distant feelings and fear, toward individuals with dementia, can lead nursing students to categorize these individuals negatively and cause them to avoid or be reluctant to provide care. Such negative emotions of caregivers can impact their interactions and effective communication with patients with dementia, potentially altering the quality of care provided. Therefore, to ensure high-quality dementia care, hospitals should foster positive emotions and attitudes towards dementia, thus calling for education programs that can promote changes in behavior.

The third category, “Efforts made regarding dementia”, consisted of the theme clusters “Strategies to address dementia” and “Hopes”. As nursing students, the participants articulated strategies for overcoming dementia by categorizing their roles, individuals with dementia, and society, similar to the findings of a previous study on nursing students and nurses [22]. As future nurses, the participants recognized that they would encounter an increasing number of individuals with dementia in various nursing settings and must be equipped with strategies to provide them with high-quality care. For this purpose, they recognized a need for specialized, comprehensive education for nursing students, including interventions and nursing care tailored to the different behavioral and psychological symptoms of dementia and systematic education based on the stages of dementia.

Furthermore, the participants expressed hopes for increased caregiving services for individuals with dementia and their families, as well as for the possibility of future treatments for dementia. South Korea became an aged society in 2017, and the prevalence of dementia has been consistently rising, with projections estimating 1 million patients in 2024 and 2 million patients by 2039. Thus, with the recognition that dementia is not “someone else’s problem”, the government has been pushing for various social care services and laying the groundwork for a culture of preparing for and preventing dementia.

Additionally, with the country’s imminent designation as a super-aged society in 2030, South Korea has encouraged the development of caregiving robots for dementia care, such as “Mario” and IoT devices for dementia prevention [23]. Moreover, integrated cognitive rehabilitation, social support, art education, and dance programs, as well as plans to expand specialized nursing homes for patients with severe dementia, are underway. These initiatives are expected to significantly alleviate the burden on individuals with dementia and their families.

The final category, “Dementia as a nursing student”, included the theme clusters of “Sense of duty” and “Need for education”. The participants expressed the need to provide the best possible care to individuals with dementia, akin to caring for a family member, and they also highlighted the need for specialized, multifaceted education in caregiving, aligning with findings from previous studies targeting nursing students and nurses [22]. Recent systematic reviews [24,25] have demonstrated that person-centered dementia care based on empathy not only improves the quality of life for individuals with dementia by alleviating neuropsychiatric symptoms but also positively impacts caregivers’ job satisfaction, stress, and burnout. Empathy refers to understanding patients’ experiences, conveying this understanding, and providing problem-solving resources based on this understanding [26]. Empathy is crucial to forming positive nurse–patient and therapeutic relationships and offering better care [27,28]. Empathy is a key competency for nursing students and an important clinical indicator in patient care [29]. Empathizing with patients with dementia is vital for nurses to actively infer patients’ mental states and engage in therapeutic interactions aimed at their comfort [30]. Therefore, educational programs on dementia for nursing students should be designed to provide person-centered dementia education based on empathy instead of simply imparting knowledge.

Additionally, the participants indicated the need for concrete and specialized education in dementia care, consistent with previous findings [19,22]. These education programs for nursing students should encompass practical training based on real-world scenarios, including the different stages of dementia and care for community-dwelling and institutionalized individuals with dementia.

Dementia cannot be completely prevented, but the risk of dementia can be minimized by managing risk factors [31]. Despite significant research on dementia, a groundbreaking cure has not yet been developed; therefore, it is important to delay the onset of dementia by reducing its risk factors and strengthening early dementia screening [32]. Accordingly, the World Health Organization (WHO) [33] emphasizes that improving dementia awareness and education should be prioritized to prevent dementia and delay its progression [31,32]. Therefore, it is necessary to develop direct and indirect nursing interventions to prevent dementia among older adults, including educational programs that can reduce controllable dementia risk factors among patients with dementia. Furthermore, as the results of this study evidence, improving positive awareness of dementia among nursing students, who are future medical professionals, may delay the onset of dementia.

## 5. Conclusions

This study employed a phenomenological approach to explore nursing students’ perceptions of dementia. Four categories, nine theme clusters, and 28 formulated meanings emerged. The four categories were “View of dementia”, “Feelings about dementia”, “Efforts made regarding dementia”, and “Dementia as a nursing student”.

This study revealed that nursing students perceived dementia through negative viewpoints and emotions, such as illness, suffering, distance, fear, and feeling sorry. However, they also demonstrated an awareness of strategies to address dementia and carried hopes for the future, along with their role in caring for individuals with dementia. These findings indicate that while nursing students may display negative perceptions and emotions about dementia, as evidenced by the identified themes, they also strive to discover strategies to address dementia, have hope for their practice, and recognize the care they must provide for patients with dementia.

The findings of this study suggest the need to develop and implement a program for improving nursing students’ positive perceptions and attitudes toward patients with dementia. Moreover, they suggest the need for multifaceted and dementia-specific nursing intervention education based on empathy for dementia from nursing students. Finally, considering that dementia awareness may vary by general characteristics, such as gender, age, and grade, and various other factors, this study recommends continued research to analyze specific influencing factors.

## Figures and Tables

**Table 1:** Classification of nursing students’ perceptions of patients with dementia.

Category	Theme Cluster	Formulated Meaning
1. View of dementia	1.1. Illness 1.2. Suffering	· A bad disease that should never be contracted · Terminal illness · Brain disease · Loss of human dignity · Destruction of the family · Economic problems
2. Feelings about dementia	2.1. Distant feeling 2.2. Fear 2.3. Feeling sorry	· It has nothing to do with me · Something that can never happen · Ambiguity about dementia · Do not remember · Abnormal behavior · Becoming an old person · Becoming a dementia caregiver · Experience as a third party with dementia patients · Experience as a third party with dementia caregivers · My discovery of becoming negative
3. Efforts made regarding dementia	3.1. Strategies to address dementia 3.2. Hopes	· The patient’s role in preventing dementia · My role in dealing with dementia patients · Society’s role in dealing with dementia patients · Various social care services for dementia patients · Possibility of future treatment for dementia patients · Expansion of family care services for dementia patients
4. Dementia as a nursing student	4.1. Sense of duty 4.2. Need for education	· Providing care like my grandparents · Providing the best care · Providing care with a tireless heart and sincerity · Need for specific and professional education about dementia · Need for education in effective nursing care · Need for training in psychology

## Data Availability

The data presented in this study are available on request from the corresponding author. The data are not publicly available owing to the information contained that could compromise the privacy of research participants.
